# Reported adverse events during out-of-hospital mechanical ventilation and ventilatory support in emergency medical services and critical care transport crews: a systematic review

**DOI:** 10.3389/fmed.2023.1229053

**Published:** 2023-10-09

**Authors:** Ricardo Sabastian Pinto-Villalba, Jose E. Leon-Rojas

**Affiliations:** ^1^Carrera de Atención Prehospitalaria y en Emergencias, Universidad Central del Ecuador, Quito, Ecuador; ^2^Facultad de Medicina, Carrera de Atención Prehospitalaria y en Emergencias, Universidad UTE, Quito, Ecuador; ^3^Medignosis, Medical Research Department, Quito, Ecuador; ^4^Escuela de Medicina, Universidad de las Américas (UDLA), Quito, Ecuador

**Keywords:** mechanical ventilation, EMS, critical care transport, complications, adverse event

## Abstract

**Background:**

Emergency medical services (EMS) and critical care transport crews constantly face critically-ill patients who need ventilatory support in scenarios where correct interventions can be the difference between life and death; furthermore, challenges like limited staff working on the patient and restricted spaces are often present. Due to these, mechanical ventilation (MV) can be a support by liberating staff from managing the airway and allowing them to focus on other areas; however, these patients face many complications that personnel must be aware of.

**Aims:**

To establish the main complications related to out-of-hospital MV and ventilatory support through a systematic review.

**Methodology:**

PubMed, BVS and Scopus were searched from inception to July 2021, following the PRISMA guidelines; search strategy and protocol were registered in PROSPERO. Two authors carried out an independent analysis of the articles; any disagreement was solved by mutual consensus, and data was extracted on a pre-determined spreadsheet. Only original articles were included, and risk of bias was assessed with quality assessment tools from the National Institutes of Health.

**Results:**

The literature search yielded a total of 2,260 articles, of which 26 were included in the systematic review, with a total of 9,418 patients with out-of-hospital MV; 56.1% were male, and the age ranged from 18 to 82 years. In general terms of aetiology, 12.2% of ventilatory problems were traumatic in origin, and 64.8% were non-traumatic, with slight changes between out-of-hospital settings. Mechanical ventilation was performed 49.2% of the time in prehospital settings and 50.8% of the time in interfacility transport settings (IFTS). Invasive mechanical ventilation was used 98.8% of the time in IFTS while non-invasive ventilation was used 96.7% of the time in prehospital settings. Reporting of adverse events occurred in 9.1% of cases, of which 94.4% were critical events, mainly pneumothorax in 33.1% of cases and hypotension in 27.6% of cases, with important considerations between type of out-of-hospital setting and ventilatory mode; total mortality was 8.4%.

**Conclusion:**

Reported adverse events of out-of-hospital mechanical ventilation vary between settings and ventilatory modes; this knowledge could aid EMS providers in promptly recognizing and resolving such clinical situations, depending on the type of scenario being faced.

## Introduction

Emergency medical services and critical care transport crews are constantly facing critical patients who need ventilatory support for acute respiratory failure (ARF), airway protection in traumatic injuries, and high levels of sedation (1–3). The survival rate and favourable outcomes depend on the capacity to maintain physiologic end-tidal carbon dioxide (ETCO2) and oxygen levels and appropriate tidal volumes (1, 4, 5). The current methods used to achieve those goals in a prehospital setting include a bag-valve-mask device (BVM) and mechanical ventilation (MV).

Manual ventilation with a BVM is typically used for its dynamic and inexpensive applications; its use also requires less training when compared to MV – which can be complex and expensive (1). However, in an out-of-hospital setting (prehospital and interfacility), where limited staff, space, and equipment are common, MV can become a great ally, freeing the provider to attend to other urgent necessities (4). BVM and MV were considered equivalent for many years (6), but the reality is different; manual ventilation can maintain a physiologic ETCO2 only 16.7% of the time and provides inappropriate vital volumes – sometimes resulting in barotrauma (1, 7).

Mechanical ventilation through the means of either an instrument inside the trachea, named invasive mechanical ventilation (IMV), or without endotracheal devices, named non-invasive mechanical ventilation (NIMV), has demonstrated effective outcomes (8, 9). NIMV is useful in conscious patients who can maintain airway patency and is indicated mainly for chronic obstructive pulmonary disease (COPD), asthma exacerbations, and acute pulmonary oedema; it works by providing positive inspiratory pressure and maintaining end-expiratory pressure (PEEP), resulting in reduced inspiratory muscle work and fatigue (10). The main modalities used are continuous positive airway pressure (CPAP) or bilevel inspiratory positive airway pressure (BiPAP), which have shown reduced mortality and intubation rates in acute respiratory failure (11).

IMV is indicated when NIMV and other therapies have failed or when advanced airway management is needed to maintain patency and support severe injuries or illnesses (i.e., when controlled pulmonary pressures and specific parameters are needed) (1, 10). In contrast with NIMV, exact target parameters can be met by controlling pressure and/or volume and by using available pre-programmed modes (10). However, management of a mechanical ventilator requires specialized training and has a steep learning curve due to the high morbidity and mortality that ensue if the ventilatory settings are incorrect or monitoring is not done properly (1, 12). Patients can be exposed to iatrogenic ventilator-induced lung injury and disturbances in blood gases (i.e., hypocapnia, hypercapnia, hypoxia, and hyperoxia), especially when the aforementioned training is not done properly (10, 12).

Knowledge of these complications as well as the ventilatory modes used in an out-of-hospital setting can be helpful for EMS and critical care transport crews; such information can enable them to reduce mortality, identify the main ventilatory settings used in the field for mechanical ventilation, and anticipate the main adverse events that can arise during the procedure, reducing morbidity.

## Methods

### Protocol and registration

This study was made following the Preferred Reporting Items for Systematic Reviews and Meta-Analyses (PRISMA) 2020 guidelines. The research protocol as well as the full search criteria were uploaded and registered in PROSPERO (CRD42021279433).

### Eligibility criteria

The language of the included studies was limited to English and Spanish. The eligibility criteria used can be found in Table 1.

**Table 1:** Summary of inclusion and exclusion criteria using the PICO framework.

	Inclusion	Exclusion
Patient		
	Adult human population (≥18 years old) Any ventilatory problem	Pediatric population (age <18 years old) Animal or manikins
Intervention		
	Out-of-hospital mechanical ventilation (prehospital or interfacility transfer) Any ventilatory mode	In-hospital mechanical ventilation Valve type ventilators like Boussignac or Vortran devices ECMO use
Comparison		
	Not applicable	Not applicable
Outcome		
	Mortality (in-hospital mortality, out-of-hospital mortality) Safety and associated problems during mechanical ventilation	
Study Design		
	Original studies	Literature reviews studies with less than 10 participants letters to the editor animal or biomechanical studies

All available studies, from the inception of the databases to July 28, 2021 were included.

### Information sources

Studies were identified in PubMed, Scopus, and Biblioteca Virtual en Salud (BVS); the references of selected articles were also screened, and only one additional reference was extracted (13). No filters were used. BVS includes scientific literature from Latin America and the Caribbean, mainly written in Spanish; inclusion of such studies is limited in most systematic reviews that only consider the English language and therefore neglect literature from the Latin-American region, where traumatic events are highly prevalent.

### Search

A search protocol was established, which can be found in the aforementioned PROSPERO registration. Search terms were tailored based on the PICO framework and the eligibility criteria; the following key terms, with variations, were used: mechanical ventilation, ventilation, out-of-hospital, mortality, safety, and adverse events.

### Study selection

Two blinded reviewers conducted an independent and uniform evaluation of the chosen studies. Deduplication was performed automatically using Mendeley Reference Manager and two stages of screening were performed. The first stage involved the screening of titles and abstracts, while the second stage involved full-text review; after these, the data of the selected studies was extracted in a spreadsheet. Any discrepancies between the reviewers were resolved by discussion and mutual consensus.

### Data items and collection process

For data extraction, we developed a spreadsheet with the following information: author, type of study, year of publication, number of ventilated patients, sex, age, type of out-of-hospital scenario (prehospital or interhospital as well as air or ground transport), ventilatory problem (traumatic, non-traumatic, and unclassified when the information provided in the study was not sufficient to classify the ventilatory problem), origin of the ventilatory problem (pulmonary, COVID-19, cardiovascular, neurologic, obstetric, septic, and other; unclassified was used when the author did not describe the origin of the problem), type of ventilation (invasive or non-invasive), mechanical ventilation mode (volume control, pressure control, pressure support, continuous airway pressure, CPAP+PS/BiPAP, and others), adverse events (divided in critical and non-critical), and mortality (divided in transport mortality and hospital mortality). If data was missing or inconsistent, we attempted to contact the authors; if they did not answer after repeated attempts, we considered it lost data. To note, due to the different illnesses of patients between out-of-hospital settings (prehospital and interfacility transport) and types of ventilation required (invasive or non-invasive) we decided to analyse them as different populations.

### Risk of bias in individual studies

Both reviewers assessed the risk of bias independently using the Study Quality Assessment Tools from the National Institutes of Health (NIH). Studies were graded as either minimally low, moderately low, or high risk of bias. If answering yes in less than 50% of the questions, the study was graded as poor and hence had a high risk of bias; if between 50 and 79%, it was graded as fair and hence had a moderately low risk of bias; and if 80% or more, it was graded as good and hence had a minimally low risk of bias, as done in a previous review (14).

## Results

**Figure 1:**
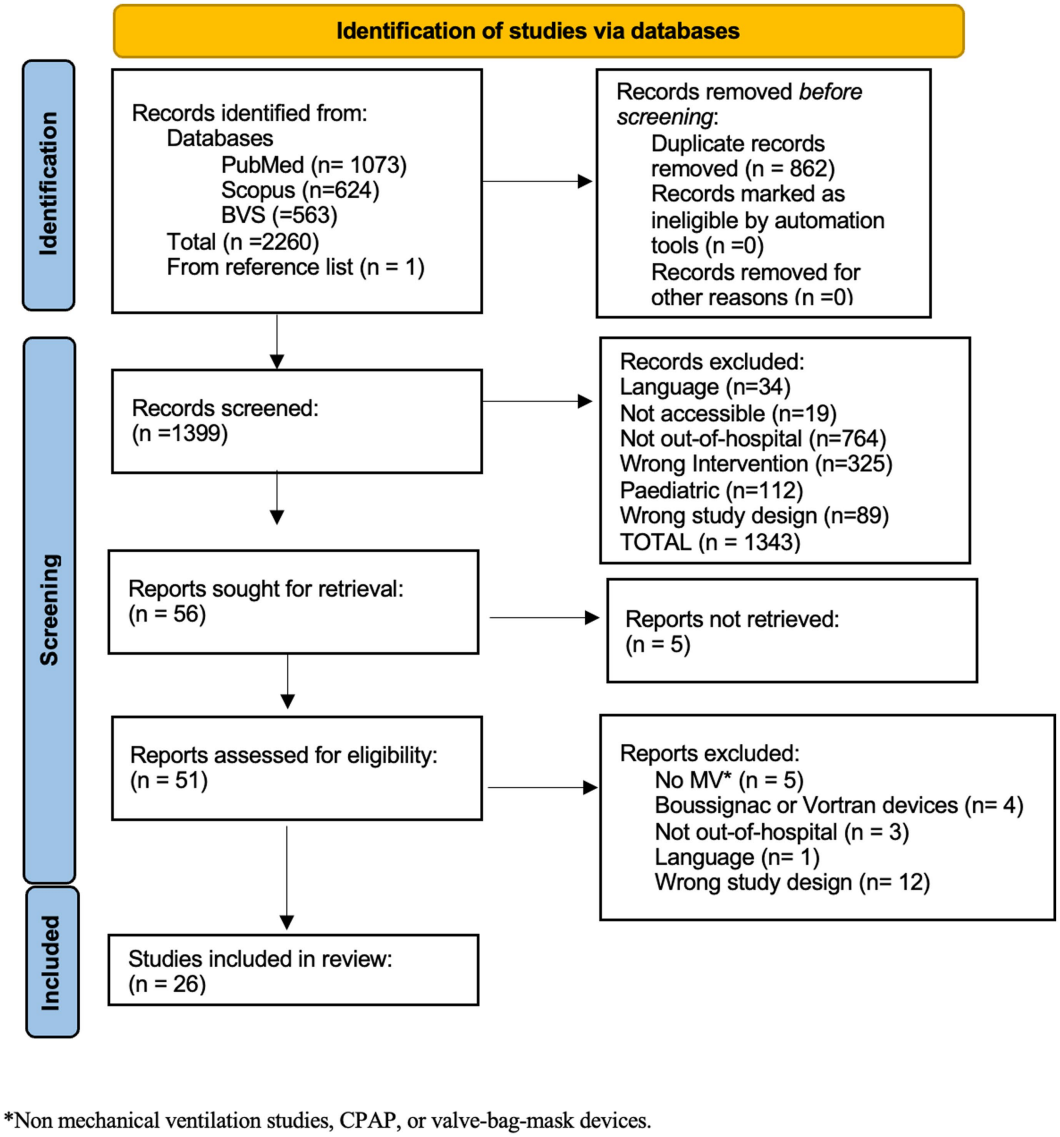
PRISMA flow chart.

The searches from the inception of the databases to July 28, 2021 yielded a total of 2,260 results. Following the removal of duplicate entries, a subset of 1,399 articles remained for further evaluation during the screening phase. The complete process of article screening can be found in Figure 1.

A total of 26 studies were finally included (3, 6, 9, 15-37); these studies, with their year of publication, design, and bias assessment, are shown in Table 2.

**Table 2:** Risk of bias assessment.

	Study	Year	Study design	Risk of bias
1	Barillo et al. (15)	1997	Cross-sectional	High Risk
2	Cheskes et al. (36)	2013	Case-control	Moderately Low Risk
3	Craven et al. (17)	2000	Randomized controlled trial	Moderately Low Risk
4	El Sayed (9)	2019	Case-control	Moderately Low Risk
5	Fuller et al. (27)	2020	Pilot randomized controlled trial	Low Risk
6	Gardtman et al. (18)	2000	Cross-sectional	Moderately Low Risk
7	Garrote et al. (20)	2015	Case-control	Moderately Low Risk
8	Gartner et al. (21)	2020	Case-control	Moderately Low Risk
9	Garuti et al. (22)	2010	Case-control	Moderately Low Risk
10	Hubble et al. (23)	2006	No randomized controlled trial	Moderately Low Risk
11	Johannigman et al. (6)	1995	Cross-sectional	Moderately Low Risk
12	Jouffroy et al. (30)	2019	Cohort Study	Moderately Low Risk
13	Jouffroy et al. (24)	2019	Cohort Study	Moderately Low Risk
14	Kallio et al. (35)	2003	Cohort Study	Moderately Low Risk
15	Kashyap et al. (26)	2016	Cohort Study	Moderately Low Risk
16	Kosowsky et al. (19)	2001	Case series	Low Risk
17	Le Cong and Robertson (28)	2013	Cross-sectional	Moderately Low Risk
18	Maddry et al. (3)	2018	Cohort Study	Moderately Low Risk
19	Michelet et al. (29)	2017	Cross-sectional	Moderately Low Risk
20	Painvin et al. (31)	2021	Cohort Study	Moderately Low Risk
21	Plaisance et al. (32)	2007	Randomized controlled trial	Low Risk
22	Roessler et al. (33)	2012	Pilot randomized controlled trial	Moderately Low Risk
23	Seethala et al. (34)	2020	Cross-sectional	Moderately Low Risk
24	Singh et al. (25)	2009	Cohort Study	Moderately Low Risk
25	Singh et al. (16)	2014	Cohort Study	Moderately Low Risk
26	Thompson et al. (37)	2008	Randomized controlled trial	Moderately Low Risk

A total of 9,418 patients who received out-of-hospital mechanical ventilation were included in our research. Among these individuals, 56.1% were identified as male. The age range of all participants spanned from 18 to 82 years. In the context of the out-of-hospital setting, 49.2% of patients received MV in a prehospital setting, while the remaining received it in an IFTS. In the prehospital setting, the prevailing ventilatory mode was found to be CPAP (NIMV), administered to a total of 2,911 patients. In contrast, volume control (IMV) was administered to 1,702 patients in the IFTS. A general summary of all ventilated patients can be found in Table 3.

**Table 3:** General results of out-of-hospital mechanical ventilation.

Data Item	*n*=	%
Patients	9,418	100%
Male	4,015	56.1%
Female	3,148	43.9%
Range of age (years)	18–82	
*Ventilatory problem*		
Traumatic	1,153	12.2%
Non traumatic	6,111	64.9%
Unclassified	2,154	22.9%
*Out-of-hospital environment*		
Prehospital	4,631	49.2%
Interfacility Transport	4,787	50.8%
Type of transport		
Air	895	9.5%
Ground	8,523	90.5%
*Ventilated patients*		
Invasive mechanical ventilation	4,880	51.8%
Non-invasive mechanical ventilation	4,538	48.2%
Adverse events during mechanical ventilation	860	9.1%
Critical events	812	94.4%
Noncritical events	48	5.6%
Mortality	791	8.4%

### Mechanical ventilation in the prehospital setting

**Figure 2:**
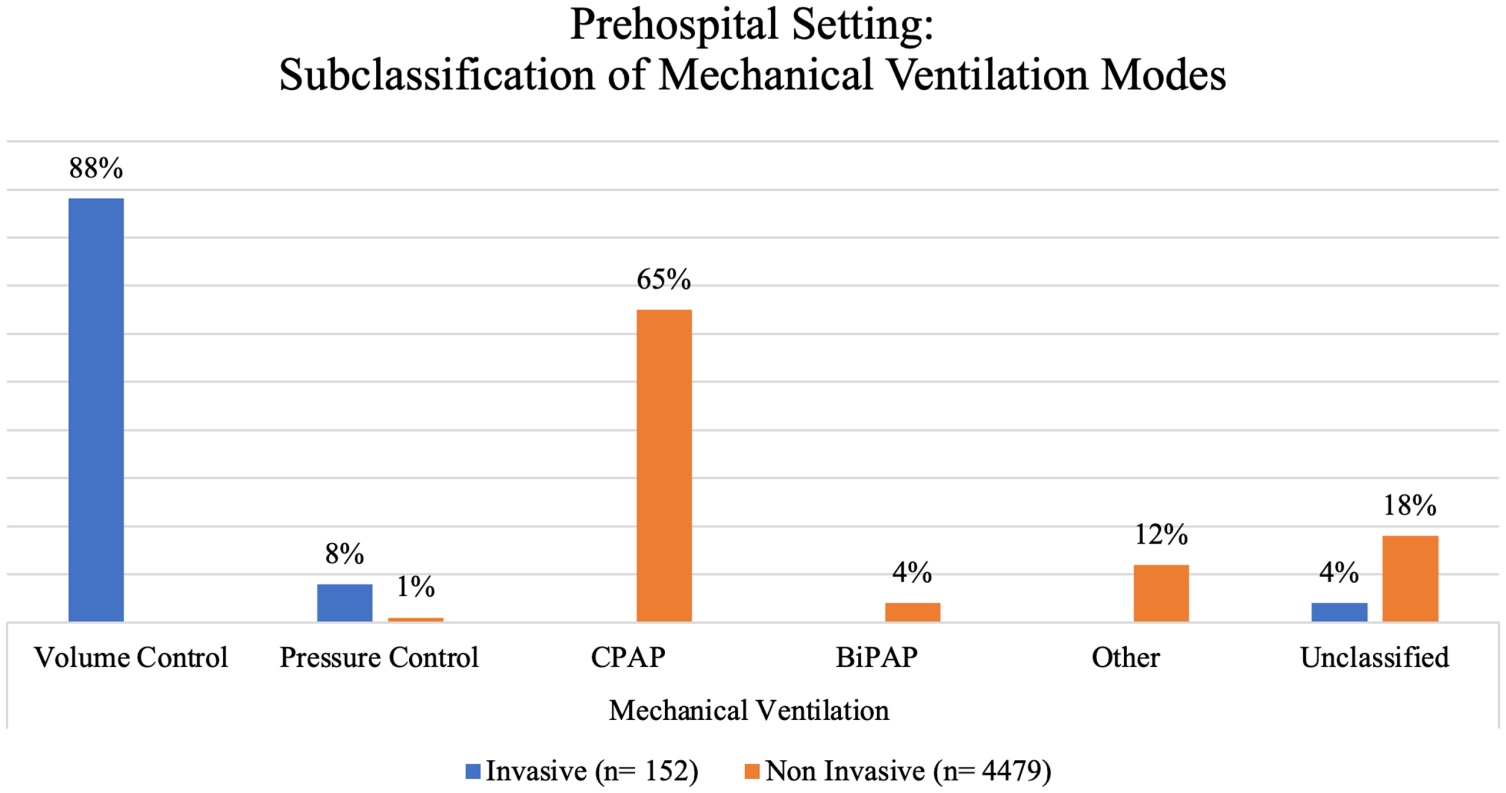
Prehospital setting: subclassification of mechanical ventilation modes.

In prehospital settings, a total of 4,631 patients underwent mechanical ventilation. Among these patients, 49% were identified as female. Notably, NIMV was the primary method of ventilatory support in 96.7% of the instances, while just 3.3% of the cases used IMV. We further subclassified these based on the ventilatory mode used (Figure 2).

**Figure 3:**
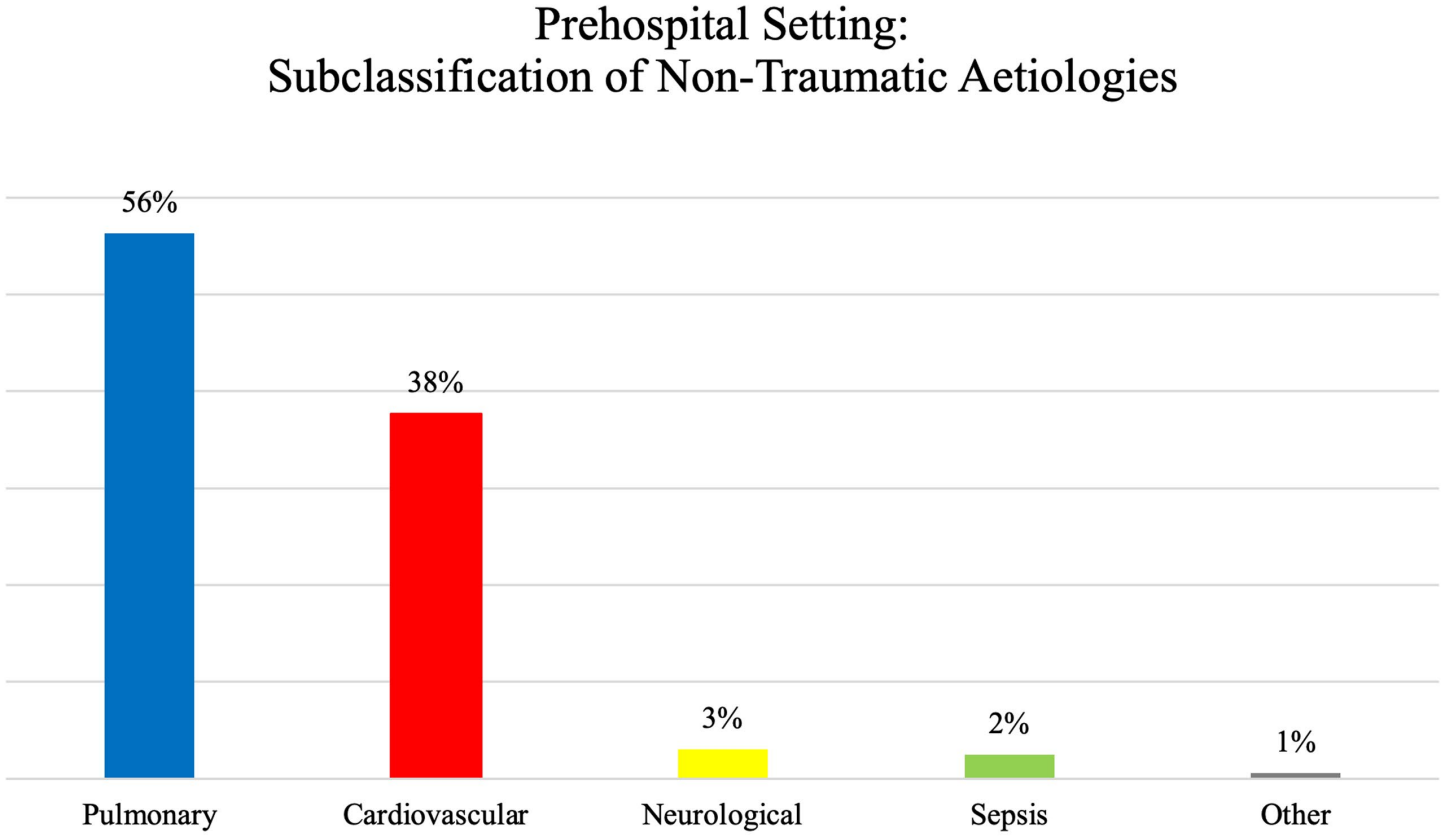
Prehospital setting: subclassification of non-traumatic aetiologies.

The analysis of the data indicates that non-traumatic ventilatory causes are more prevalent than traumatic ventilatory causes in the prehospital setting, accounting for 94.6 and 5.4%, respectively. Please see Figure 3 for the subclassification of non-traumatic aetiologies.

**Figure 4:**
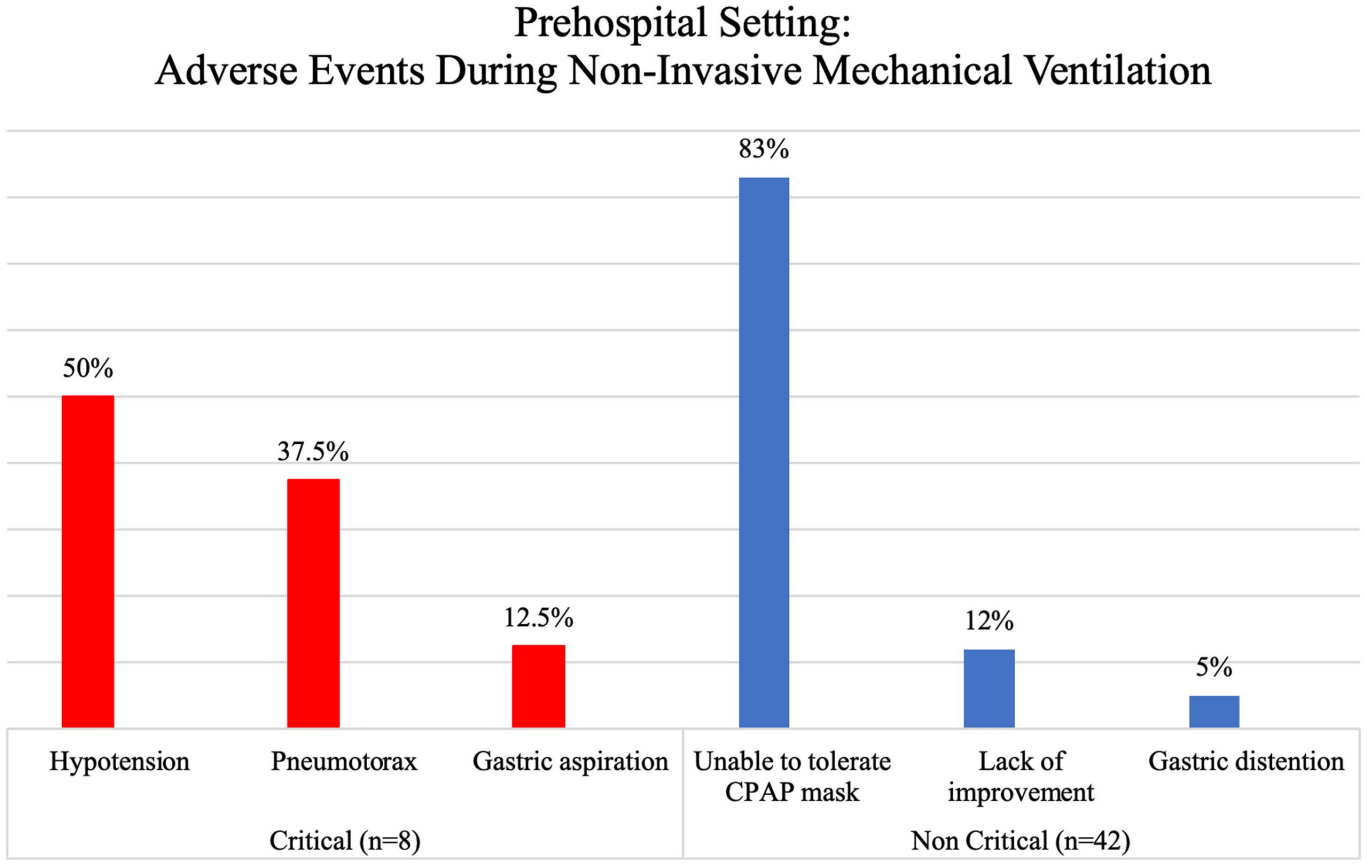
Prehospital setting: adverse events during mechanical ventilation.

Adverse events that were seen during ventilation in the prehospital setting were documented in 1.8% of patients. These events were categorized based on their potential to cause life-threatening situations, with critical events accounting for 16% and non-critical events accounting for 84% of the reported cases. It is worth noting that adverse events were only recorded in relation to NIMV, as shown in Figure 4. The most critical events seen in this study were hypotension, which occurred in 50% of cases, and pneumothorax, which occurred in 37.5% of cases. These events were predominantly observed in patients with non-traumatic injuries, namely those with cardiovascular or pulmonary illnesses.

The vast majority of pre-hospital transfers, accounting for 99.6% of cases, were conducted by ground transportation. Among these cases, 661 patients, or 14.2% of the total, were reported to have died upon admission. Notably, no fatalities were recorded during the actual transport phase in the dataset under analysis.

### Mechanical ventilation in the interfacility transport setting

**Figure 5:**
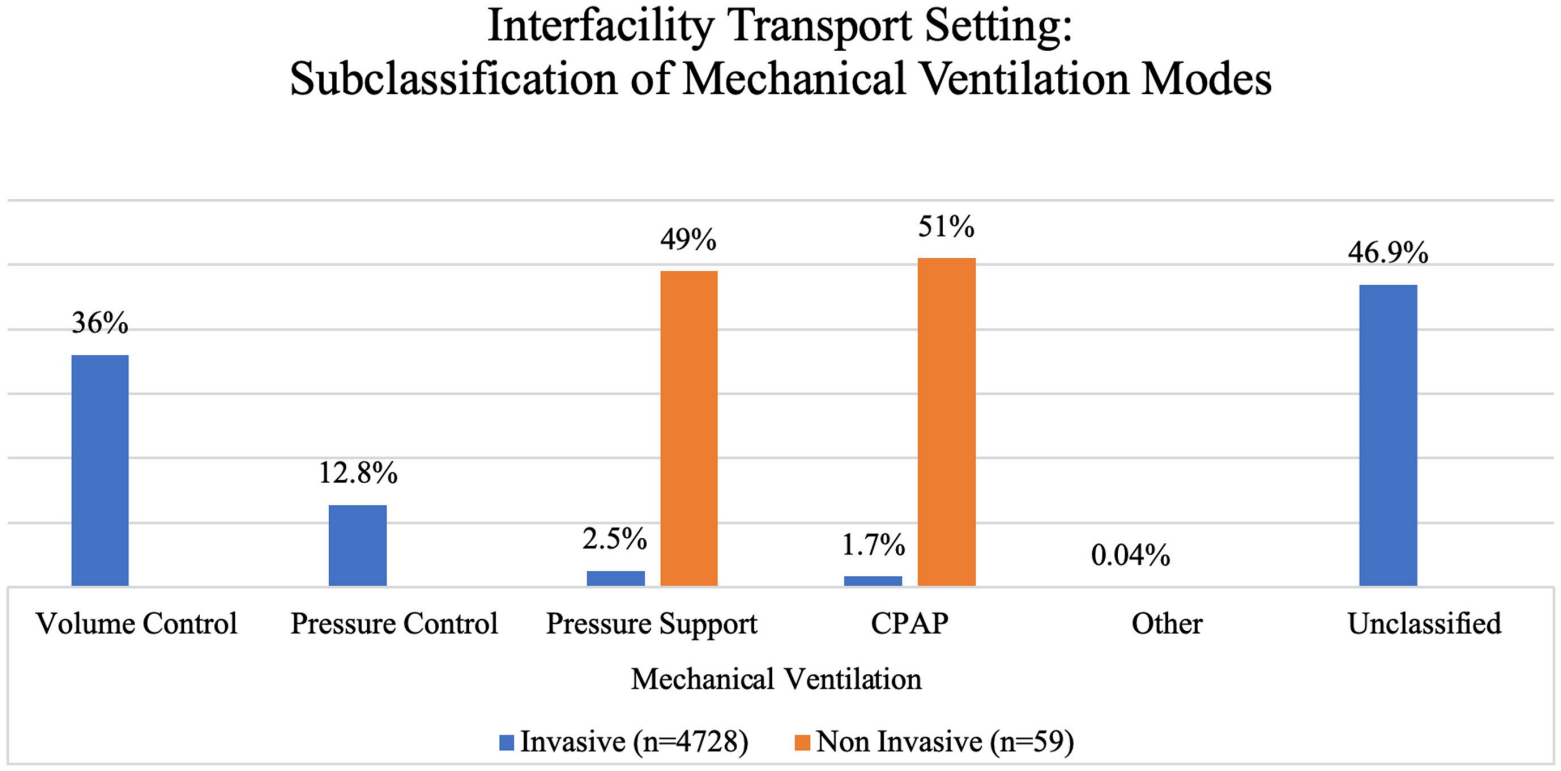
Interfacility transport setting: subclassification of mechanical ventilation modes.

In the IFTS, a total of 4,787 individuals underwent mechanical ventilation. Among these, 68% were male and the majority (98.8%) got IMV as their primary form of ventilatory support, while a smaller proportion (1.2%) used NIMV. We further subclassified these based on the ventilatory mode used (Figure 5).

**Figure 6:**
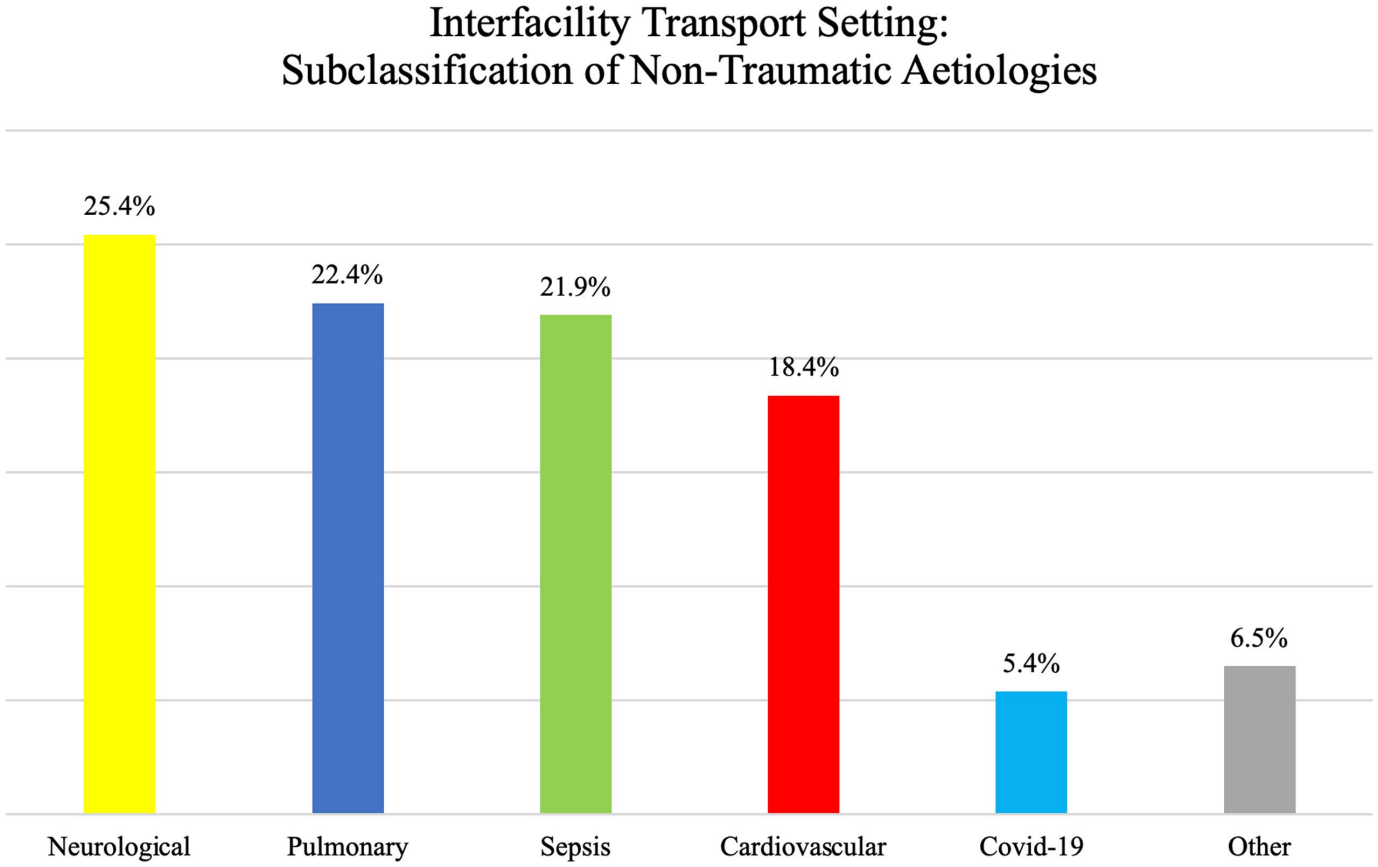
Interfacility transport setting: subclassification of non-traumatic aetiologies.

Similar to the prehospital setting, non-traumatic causes of ventilatory issues were more often seen than traumatic causes, with a prevalence of 36.1% compared to 20.8%. Please refer to Figure 6 for a breakdown of the subcategories of non-traumatic causes. Unfortunately, in this group, there was an important lack of data that prevented us from classifying the aetiology of the ventilatory problems (43.1% had to be categorized as unclassified).

**Figure 7:**
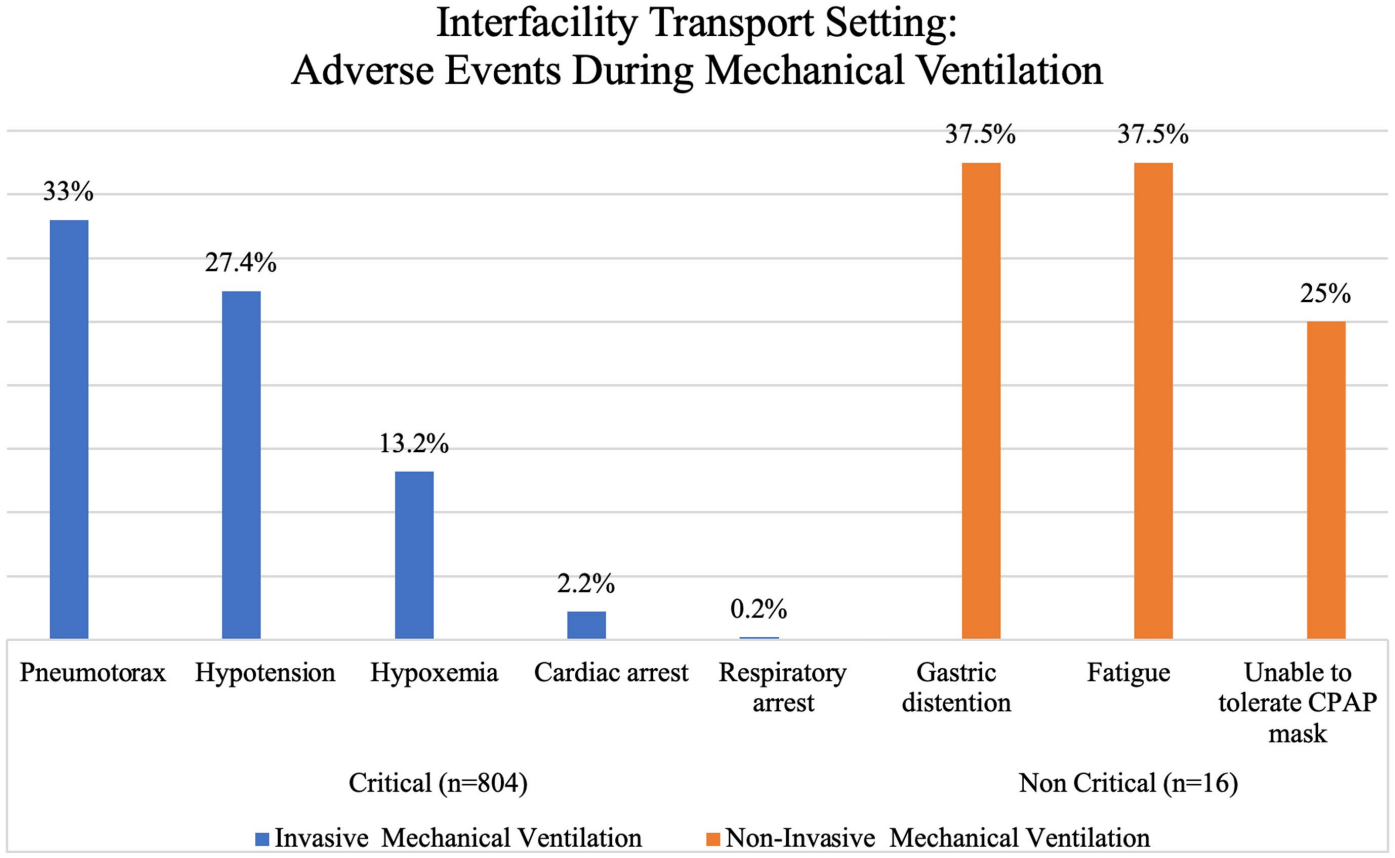
Interfacility transport setting: adverse events during mechanical ventilation.

Reported adverse events during ventilation in the IFTS occurred in 16.9% of patients. Among these occurrences, 99.3% were classified as critical, while the remaining 0.7% were considered non-critical. The majority of adverse events were recorded in patients receiving IMV, accounting for 97.5% of cases, while only 2.5% of cases were associated with NIMV. Each of these categories was further divided into specific types of events and presented in Figure 7. The two main critical events reported in this group of patients were pneumothorax (33%), mainly in traumatic illnesses (i.e., blast or penetrating injuries), and hypotension (27.4%), mainly in non-traumatic injuries (i.e., cardiovascular or pulmonary illness). It is noteworthy that despite the relatively high occurrence of these events, overall mortality remained relatively low at 3%.

The majority of interfacility transports, around 82%, were conducted via land transportation, while the remaining proportion used air transport. Among the whole population of mechanically ventilated patients in the IFTS, a mortality rate of 3% was observed upon arrival. No instances of death were recorded during the transit phase in the data that was analysed.

## Discussion

This systematic review was performed to assess the use of out-of-hospital mechanical ventilation, its features, reported adverse events, and mortality.

The attention of critically-ill patients in out-of-hospital settings is a challenge for EMS and critical care transport crews that need to expertly handle multiple complex procedures and therapies in a limited physical space with minimal equipment and staff – often following different protocols based on the institution or regulatory bodies of the country (16). The use of equipment in these instances, either at the location of attention or during transportation between centres, can help prolong life until definitive care in a hospital (16). In the course of these events, the EMS and critical care transport crews need to recognize all possible life-threatening conditions associated with the illness, as well as the adverse events that may arise from the use of devices, in order to prevent them from happening (16). However, it should be noted that our study does not establish a direct causal relationship between complications and mechanical ventilation, primarily due to limitations in the design and available information in the studies included. Nonetheless, our study does emphasize the frequency of adverse events in patients receiving mechanical ventilation. It underscores the importance of considering these complications when managing patients with traumatic or non-traumatic conditions, providing an opportunity to proactively address them based on the specific patient characteristics, the mode of mechanical ventilation employed, or the type of out-of-hospital setting.

Mechanical ventilation was typically used in hospitals, but over time this procedure became increasingly common in the prehospital setting – during transports and primary attention (6, 10, 38). In our study, we found an ample range of ages (18–82), which might be due to the fact that this procedure is being used more frequently in young people due to traumatic conditions such as burns, brain lesions, and combat injuries (3, 15), while still being beneficial for older adults that usually have chronic conditions or emergent conditions elicited by previous illnesses like acute cardiogenic pulmonary oedema or exacerbations of chronic obstructive pulmonary disease (COPD) (18, 19).

Mechanical ventilation during interfacility transport is more common than in the prehospital setting, especially during ground transport, which happens to be the most common modality due to higher availability and lower cost when compared to air ambulances (39); in our study, 90.5% were transported by ground using a variety of ventilatory modalities.

The means of mechanical ventilation were evenly distributed between invasive 51.8% (mainly in IFTS) and non-invasive 48.2% (mainly in prehospital settings). NIMV was represented primarily by CPAP (64.4%), which is not surprising given that previous studies have shown improvement on vital signs, reduction in short-term mortality, and less need for endotracheal intubation in patients with acute pulmonary oedema and chronic obstructive pulmonary disease (COPD) when using this ventilatory mode (40–42). In contrast, the main modality for IMV in out-of-hospital settings was volume control ventilation (37.6%), while the unclassified category represented 45.6% of all IMV patients. Showcasing that, studies assessing IMV in a prehospital setting often do not provide enough information to clearly determine the modality being used, leading to the loss of valuable information such as detecting common complications of each modality that might differ from those in a hospital setting. This is especially concerning when considering that the correct use of a ventilator with lung-protective parameters by properly trained prehospital professionals increases survival by improving the chances of accessing appropriate medical care (3, 24).

### Adverse events

#### Hypotension and pneumothorax

Critically ill patients can face multiple adverse events during out-of-hospital care – evidence suggests an incidence of one in fifteen transports, with hypotension being a common complication in 4.4–11.9% of cases (16, 25, 43). This contrasts with our findings that show a higher incidence of pneumothorax than of hypotension (33% vs. 27.4%) in IFTS, possibly due to the underlying condition that required ventilation in the first place; however, in the pre-hospital setting, hypotension does show a higher incidence than pneumothorax (50% vs. 37.5%). However, we could not directly correlate hypotension or pneumothorax with the use of out-of-hospital mechanical ventilation due to the lack of complete data in the available evidence that would allow us to establish causality. Regardless of the aetiology, EMS and critical care transport personnel that use mechanical ventilation in out-of-hospital settings must be on high alert for such events and be prepared to solve them when they arise.

#### Oxygen levels

Hypoxia is a common condition in the prehospital setting, often requiring ventilatory support (44–46). Although this adverse event was less common than hypotension or pneumothorax in our analysis (13.1%), the EMS and critical care transport crews need to take it into account and monitor closely the oxygen availability and consumption along the transport; an ample supply of oxygen should be available in the vehicle, especially when expecting to travel long distances.

Although hypoxia is frequently discussed in the literature, hyperoxia – which can go unnoticed – is equally harmful to the patient; hence, prehospital responders must target SpO_2_ values above 94% and below 96% (30, 47).

#### Mortality

Although our reported mortality is relatively low – 8.4% overall for out-of-hospital mortality, 3% for the IFTS, and 14.3% for the prehospital setting – it correlates with epidemiological prehospital mortality studies in airlifted and ground patients (8.2 and 14.6%, respectively) (48). Continuing education and appropriate training of responders might help in improving the safety of this procedure. We performed a sub-analysis that revealed that most of the mortality occurred in airlifted patients; however, this is more likely explained by the severity of illness (requiring faster transport through the air) than by the direct effect of the type of transport or the use of mechanical ventilation. Therefore, we encourage out-of-hospital personnel to train in the proper use of all of their available devices, including the mechanical ventilator, in order to provide the best treatment possible until definitive care can be obtained.

## Limitations

Our analysis was dependent on the quality of the data in the included studies, some of which lacked specific information that prevented an in-depth analysis of correlations or causality between mechanical ventilation and patient complications due to high heterogeneity. Regardless of comparators, when all patients who received the intervention are considered, less than 10% suffered an adverse event or manifestation of the underlying pathology associated with the condition that led to the intervention. Most of the studies analysed transported patients as a whole (i.e., including ventilated and non-ventilated), which precluded a subgroup exploration of aetiology, diagnosis, and complications or number of complications per patient of only those being mechanically ventilated; almost 22% of the patients included in this review had to be categorized as “unclassified” due to missing information. Additionally, there was little information regarding the ventilatory modes used and ventilatory settings, especially with IMV, which prevented us from properly assessing if the ventilatory modality was related to mortality or morbidity.

## Conclusion

The mechanical ventilator can be a helpful device in an out-of-hospital setting given that it liberates EMS and critical care transport personnel to provide support for the patient’s condition during the transport in different areas other than ventilation. Additionally, achieving proper ventilatory parameters – while avoiding hypoxemia, low or high tidal volumes, and hypo or hypercapnia – becomes easier and significantly superior to using the BVM device.

Adverse events in out-of-hospital mechanical ventilation vary depending on the type of out-of-hospital setting and ventilatory mode. Although adverse events around mechanical ventilation are limited, the EMS and critical care transport crews must be trained and prepared to promptly recognize them or identify conditions that can lead to them; training and continuing education are paramount to achieving this.

## Data availability statement

The original contributions presented in the study are included in the article/supplementary material, further inquiries can be directed to the corresponding author.

## Author contributions

RP-V: conceptualization, data curation, formal analysis, investigation, visualization, writing – original draft preparation, and writing – review & editing. JL-R: conceptualization, data curation, formal analysis, funding acquisition, investigation, methodology, project administration, resources, software, supervision, validation, and writing – review & editing. All authors contributed to the article and approved the submitted version.

## Abbreviations

ARF: acute respiratory failure
BiPAP: bilevel inspiratory positive airway pressure
BVM: Bag-Valve-Mask-Device
BVS: Biblioteca Virtual en Salud
COPD: chronic obstructive pulmonary disease
CPAP: continuous positive airway pressure
ETCO2: end-tidal carbon dioxide
IFTS: interfacility transport settings
IMV: invasive mechanical ventilation
MV: mechanical ventilation
NIH: National Institutes of Health
NIMV: non-invasive mechanical ventilation
PEEP: end-expiratory pressure
PRISMA: Preferred Reporting Items for Systematic Reviews and Meta-Analyses
